# High Levels of Physical Activity May Promote a Reduction in Bone Mineral Density in Peritoneal Dialysis

**DOI:** 10.3390/medicina56090464

**Published:** 2020-09-11

**Authors:** Armando Raimundo, Zelinda Charrua, Nuno Batalha, Catarina Pereira, Jose Parraca, Pablo Tomas-Carus

**Affiliations:** 1 Departamento de Desporto e Saúde, Escola de Ciência e Tecnologia, Universidade de Évora, 7000 Évora, Portugal; zelindacharrua@hotmail.com (Z.C.); nmpba@uevora.pt (N.B.); clnp@uevora.pt (C.P.); jparraca@uevora.pt (J.P.); ptc@uevora.pt (P.T.-C.); 2 Comprehensive Health Research Center (CHRC), University of Évora, 7000 Évora, Portugal

**Keywords:** body composition, bone density, muscle strength, peritoneal dialysis, physical activity

## Abstract

*Background and objectives:* Peritoneal dialysis (PD) patients are expected to present lower levels of physical activity, unhealthy changes at the body composition level, and low levels of strength. Firstly, this study aimed to report the sex differences in physical activity, body composition and muscle strength and the relations among these variables. Secondly, we analyze the relationship between physical activity and biochemical parameters. *Materials and Methods:* Thirty-four patients (13 women and 21 men) participated in this study. Body composition was assessed by bioimpedance and dual-energy X-ray absorptiometry (DXA), and maximum isokinetic unilateral strength, analytical parameters and physical activity levels were evaluated. *Results:* The men showed higher values for weight, height, lean body mass, bone mineral content, bone mineral density (BMD) and total body water, while women showed higher values for the percentage of fat mass and hydration of lean body mass (*p* < 0.05). No differences between the sexes were found in different levels of physical activity; however, males registered significantly higher values for isokinetic strength variables except for knee extensor strength. BMD was positively related to sedentary activity and negatively related to moderate and vigorous activity (*r* = 0.383 and *r* = −0.404, respectively). Light physical activity was negatively correlated with albumin (*r* = −0.393) and total protein (*r* = −0.410) levels, while moderate/vigorous activity was positively correlated with urea distribution volume (*r* = 0.446) and creatinine clearance (*r* = 0.359) and negatively correlated with the triglyceride level (*r* = −0.455). *Conclusions:* PD patients with higher levels of physical activity present better results in terms of body composition and biochemical parameters. Additional studies should be conducted to clarify the relation between physical activity level and BMD.

## 1. Introduction

Chronic kidney disease (CKD), as well as dialysis treatment itself, causes a series of systemic, metabolic and hormonal changes, factors that adversely affect the physical function, nutritional status and quality of life of patients [1]. It is known that peritoneal dialysis (PD) patients typically lead sedentary lifestyles and are usually weaker, less active and walk more slowly than controls [2]. Physical inactivity is also related to a decreased health-related quality of life, increased morbidity and increased health-care expenditure and is estimated to be the fourth leading cause of death [3]. Moreover, PD patients usually present with lower levels of strength [4] than healthy sedentary controls [5], partly due to the loss of muscle mass [6]. As muscle strength is an important predictor of gait speed in patients on dialysis [2] and likely to be a major determinant of physical functioning and thus quality of life, it is important to counteract the decrease in strength by implementing exercise programs [1].

Compared with healthy individuals of the same age and sex, PD patients showed no significant differences in terms of body composition [1]. Dual-energy X-ray absorptiometry (DXA) measures the distribution of bone mineral, fat, and lean body mass (LBM), and it is considered to be better than other noninvasive methods for determining body composition in PD patients [7]. Regarding bone mineral density (BMD), PD patients present values similar to those of normal subjects [8].

Although some studies report that increased physical activity (PA) levels in patients with PD can generate benefits in controlling increased cardiovascular morbidity, mortality and loss of function, the effects of PA in patients on PD remain doubtful [9]. Recently practitioners have suggested that PD patients should increase their PA levels, as this increase allows patients to improve their quality of life and to reduce the adverse effects of inactivity [10].

Considering the importance of PA, the aim of this study is to investigate sex differences in PA, body composition and muscular strength; to examine relations between PA level, body composition and muscle strength; and to study the relationship between PA and biochemical parameters in patients undergoing regular PD. To our knowledge, there have been no publications on the differences between the sexes and the relationships between the variables described above. 

## 2. Materials and Methods

### 2.1. Patients

PD patients were recruited from the Nephrology Unit of the Espírito Santo Hospital of Évora, Portugal. The eligibility criteria for the present study were as follows: age 18 years and over; a history of established PD for at least six months; the absence of acute or chronic medical conditions that could interfere with the collection of data for the outcome measure; with the permission of the physician, independent ambulation for more than 100 m with or without an assistive device; no cognitive limitations; stable condition; do not suffer from any comorbidity that may influence the study variables; and willingness to participate. All the PD patients were treated with bicarbonate/lactate-mix-buffered PD fluids.

In total, 49 PD patients from the Alentejo region were eligible and invited to participate of whom 34 patients (13 women and 21 men) agreed to participate. Of the 13 women in the study, four were postmenopausal.

All participants were informed about the objectives of the study and gave their informed consent to participate.

The study was approved by the University of Évora Ethics Committee (with code of ethical approval number 18,040) and conducted in accordance with the World Medical Association’s Declaration of Helsinki [11].

### 2.2. Data Collection

The study protocol included assessments of body composition (bioimpedance/DXA), maximum isokinetic unilateral strength, analytical parameters (blood count, hematocrit, total cholesterol, high-density lipoprotein (HDL), low-density lipoprotein (LDL), parathormone (PTH), urea and creatinine), clearance creatinine (CCR), PD treatment adequacy (KT/V) and residual renal function (RRF), and characterization of physical activity level. The study participants were asked to attend the Nephrology Unit of the Espírito Santo Hospital of Évora to perform all study-related tests except the body composition and isokinetic strength tests, which were performed at the Health and Sports Department of the University of Évora The same research staff members performed the assessments for all subjects using the same equipment to reduce the risk of bias. On the testing day, a fasting blood sample, the last 24 h of drained liquid, and total urine volume over the last 24 h were collected. To eliminate the possible influence of intraperitoneal volume on exercise capacity as well as on body composition variables, the patients underwent testing after total peritoneal fluid drainage.

### 2.3. Anthropometric Measurements

Body height and weight were determined by standard anthropometric methods. Height was measured to the nearest mm while the participant was barefoot or wore stockings and stood upright against a Holtain portable stadiometer. Weight was measured to the nearest 0.10 kg with the participant lightly dressed (underwear and t-shirt) using a portable digital scale (Tanita TBF 300, Tanita Corp., Arlington Heights, IL, USA).

Body mass index (BMI) was calculated as weight/height^2^ (kg/m^2^).

### 2.4. Bioimpedance Measurements

Total body water (TBW) and impedance were assessed using a bioelectrical impedance analyzer (Tanita TBF 300, Tanita Corp., Arlington Heights, IL, USA) based on the principle of single-frequency leg-to-leg bioelectrical impedance, applied to the body via four electrodes fixed to a measuring platform positioned at predetermined points on the soles of the feet. This apparatus uses an electrical current of low amplitude (500 μA) and low frequency (50 kHz). Participants wiped their feet on a towel prior to stepping on the scale. Data collection was performed at the same time of the day in all cases to allow for the same conditions.

The percentage of hydration of lean mass (%HLBM) was calculated using the TBW obtained by the Tanita device and the LBM acquired by DXA according to the following equation [12]:

*%HLBM = (TBW/LBM)* · 100

### 2.5. Body Composition

Body fat mass (FM), percentage of fat mass (%FM), BMD, bone mineral content (BMC), LBM and fat-free mass (FFM), which represents the sum of the LBM and BMC, were assessed by dual-energy X-ray absorptiometry (DXA, Hologic QDR, Hologic, Inc., Bedford, MA, USA). DXA uses a three-compartment model to quantify FM, LBM, and BMC and is currently considered the most practical means to determine the body composition in PD patients [7].

### 2.6. Muscle Strength

Maximal unilateral isokinetic strength was measured in the knee extensors/flexors and in the elbow extensors/flexors during concentric actions at 60°/s (3 reps) using an isokinetic dynamometer (Biodex System 3, Biodex Corp., Shirley, NY, USA).

All the participants were seated on the dynamometer such that the axis of the dynamometer coincided with the axis of the knee or elbow (depending on whether leg or arm strength was being evaluated), according to the standards provided in the Biodex Isokinetic Manual [13]. Adjustments of the seat position were recorded for each subject to maintain standard positions. At the start of each single test, the participant was asked to relax so that the passive effect of gravity on the limb could be registered. Every test was performed using a hard deceleration cushion. Verbal encouragement was provided as stimulation for the subject to exert maximal effort. The range of motion assessed was between 100° and 0° at full leg extension to evaluate the lower limbs and between 135 and 0° at full arm extension to evaluate the upper limbs, allowing determination of the preload activation moment, which cannot be obtained during eccentric movements. Prior to effective measurement, all subjects were allowed to perform three trials of the requested movement at moderate intensity for familiarization. The highest correct gravity peak moment value (peak torque) was registered after standard computer adjustments were made and recorded for further analysis.

### 2.7. Physical Activity

Physical activity was assessed by accelerometry (ActiGraph, model GT1M, Fort Walton Beach, Florida). The accelerometer had a reduced dimension (3.8 × 3.7 × 1.8 cm, 27 g). All participants were asked to wear the accelerometer on the right hip near the iliac crest for seven consecutive days. Delivery and reception of the accelerometer to the participants, as well as the explanation of its use, were always performed personally by the same research staff member [14]. All devices were activated on the first day at 6.00 a.m., and data were recorded in 15-s intervals, because 1-min intervals can potentially result in underestimation of the participation in moderate- or high-intensity physical activity [15], as individuals in this population have a lower level of daily activity than healthy sedentary individuals. The accelerometer was activated and data downloaded using ActiLife Lifestyle software (v.3.2). The data were processed with MAHUffe v.1.9.0.3 (available at www.mrc-epid.cam.ac.uk) using the original downloaded files (*. dat). For the analyses, a valid day was defined as the monitor having been worn for 500 or more minutes (8 h and 20 min), which is less than the minimum duration of daily accelerometer use suggested by Ward et al. [14], since these patients spend several hours immobilized in dialysis. Because individuals’ weekday and weekend activity patterns differ, it has been recommended that weekdays and weekend days are combined in measurement protocols [15]. Accordingly, we collected data for six consecutive days, including two weekend days. All subjects complied with the requests and were thus included in the data analysis.

The amount of activity, as assessed by accelerometry, is expressed as the number of minutes per day a participant spent performing physical activity of different intensities [16] and the number of steps he or she took per day. In the present study, we used the cut-offs suggested by Troinano et al. (sedentary activity 0–99 counts; light activity 100–2019 counts; moderate activity 2020–5998 counts; vigorous activity higher than 5998 counts) [16].

### 2.8. Analytical Chemistry

A 15 mL blood sample was collected for assessment of baseline serum creatinine and glucose concentrations.

The total volumes of urine and drained liquid over 24 h were recorded for entry into the PD ADEQUEST computer application Renal Soft, as were urea, creatinine and glucose levels to calculate the Kt/V and CCR. All the data were entered into the above program, allowing easy calculation of Kt/V and weekly CCR in L/1.73 m^2^. The CCR was normalized to a body surface area of 1.73 m^2^ [17].

### 2.9. Statistical Analysis

The group data are represented as the mean (standard deviation) unless otherwise stated. All data were tested for normality using the Shapiro-Wilk test because the groups were small. When examining differences within the study population, the independent Student’s t test was applied for data with a normal distribution; for the non-normally distributed data, the Mann-Whitney U test was used to determine differences between the means. When we used the *t* test, the *p* value was corrected when heterogeneity of variance was evident. Correlation analysis was performed using Spearman rank correlation.

The minimum sample size representative of the PD patient population of Alentejo (*n* = 49) was calculated by the epidemiologic statistical OpenEpi software [18] to be 44 with a 95% confidence interval, and 38 with an 80% confidence interval.

## 3. Results

Participant characteristics, such as demographics, body composition, physical activity, muscle strength, and laboratory variables, are presented in Table 1.

There was a significant difference between men and women in weight, height, %FM, LBM, BMC, BMD, TBW and %HLBM (Table 2). Men showed higher values for weight, height, LBM, BMC, BMD and TBW, while women showed higher values for %FM and %HLBM.

Physical activity data according to sex are presented in Table 3, including values for sedentary, light, and moderate/vigorous physical activity. No differences in time spent at the various levels of physical activity were found between the sexes. However, regarding muscle strength, males registered significantly higher values for all variables except for knee extensor muscle strength (Table 3).

**Figure 1:**
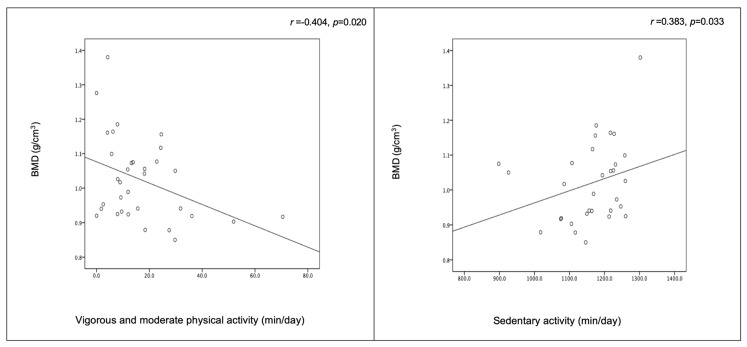
Relationship between physical (in)activity and body composition.

Figure 1 shows a negative correlation between vigorous/moderate physical activity and BMD, as well as a positive correlation between sedentary activity and BMD (*r* = −0.404 and *p* = 0.020 and *r* = 0.383 and *p* = 0.033, respectively).

**Figure 2:**
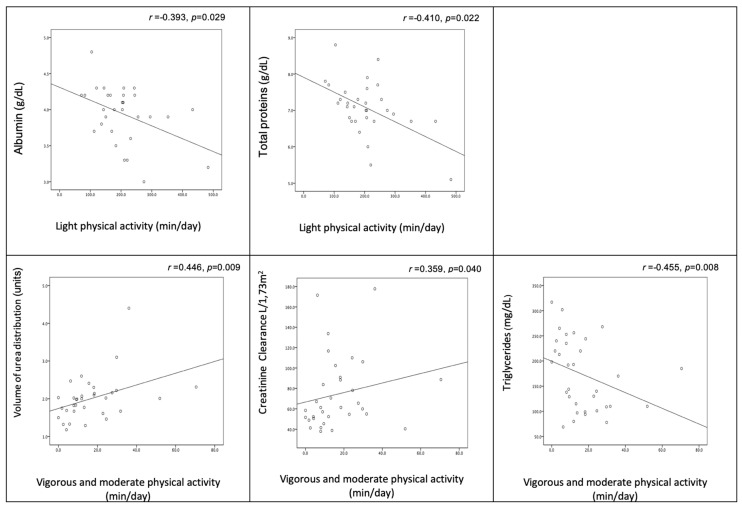
Relationship between physical (in)activity and biochemical indicators.

Figure 2 shows the significant correlations between physical (in)activity and biochemical indicators. Light physical activity was negatively correlated with albumin and total protein levels, while moderate/vigorous activity was positively correlated with volume of urea distribution and creatinine clearance and negatively correlated with triglyceride level.

## 4. Discussion

The first purpose of this study was to investigate sex differences in body composition, physical activity and muscular strength. Sex differences were expected in height, weight, %FM, LBM, BMC, BMD and %HLBM (Table 2) among PD patients, as these differences have been demonstrated in healthy subjects [19]. It seems that dialysis treatment does not affect sex differences in body composition, with men presenting higher LBM values and women presenting higher %FM values [20].

The age, height, weight and BMI values reported in the present study are similar to those reported in previous studies [1]; however, our values for FM are relatively high. As explained by Fernstrom et al. [21], the time in PD increases FM. One possible explanation for observed FM values observed in the present study being higher than those reported in other studies may be the differences in the duration of dialysis, since the subjects in this study were treated with dialysis for at least six months, while those included in the study by Medici et al. [22] were treated for a median of only four months. However, it is important to note that the results of the PD patients were different from those of healthy subjects. Particularly, PD patients had lower lean mass values. Baxman et al. [23] found that lower physical activity levels are associated with lower muscle mass values. We further emphasize that the values reported in this study in PD patients are even lower than those reported in previous studies in PD patients [24]. This finding is relevant since it is known that mortality risk in these patients increases as LBM (sarcopenia) and consequently BMI and weight decrease [25]. Physical activity and exercise programs seem to be important for preventing or ameliorating muscle atrophy in these patients, as there are limited pharmacological interventions for muscle atrophy [26].

An appropriate body composition seems to be positively associated with physical activity in PD [4,27], which, as discussed below, is a problem in these patients due to reduced levels of PA. As mentioned by Cupisti et al. in their clinical setting, implementing regular physical activity could contribute to maintaining muscle mass and increasing energy expenditure [27]. This outcome may help PD patients, as they present lower LBM values and higher %FM values (particularly women).

Regarding physical activity (Table 3), sex differences were not found. Both sexes registered approximately 5000 steps, which is 1000 steps more than the number reported by Broers et al. [28] in a population similar to that included in the present study. However, we highlight that the number of steps registered was significantly lower in the PD patients than in the healthy subjects [27,28], which contributes to disability and a poor nutritional status, thereby increasing the risk of morbidity and mortality [27]. Moreover, as suggested by Broers et al. [28], the cohort included in the present study should be considered sedentary, as they exhibited a sedentary lifestyle, which has recently been defined as fewer than 7500 steps being taken per day.

Sex differences were found in terms of isokinetic strength, even when we normalized the force values to body weight (Table 3), in knee flexion and in upper limb extension and flexion. These results are consistent with those reported by Blake and O’Meara [29], although the angular velocities were different (in the present study, an angular velocity of 60°/s was used, while in the aforementioned study, an angular velocity of 180°/s was used), further highlighting the fact that the patients in our study presented lower values than did healthy subjects. As expected, men presented higher LBM values (Table 2), which are likely to contribute to high muscle strength [30]. As mentioned above, since these patients lose muscle mass, in the isokinetic strength test they also show values lower than those of healthy subjects [31]. Structural changes in muscles occur over time in these patients, leading to changes such as reduced muscle strength and endurance which influence their quality of life, emphasizing the need for exercise programs tailored to the needs of these patients [32].

The second purpose of this research was to examine the relationships among physical activity, body composition and muscle strength in patients undergoing regular PD. Our results show that components of body composition, as assessed by DXA, have limited associations with physical activity as well as with muscular strength.

In fact, the main finding of the present study was the negative relationship between physical activity and BMD. Several studies have reported that higher levels of physical activity are related to higher values for bone-related variables in PD patients as well as in healthy people [33]. However, our results showed the opposite result. The level of physical activity performed daily by these patients was very low, even lower than the recommended levels [34]. These recommendations reported by several national and international institutions suggest that adults and aging people should complete at least 30 min of moderate/vigorous physical activity every day and take more than 7500 steps per/day [35]. In agreement with the suggestions of Inker et al. [36], the Kidney Disease Improving Global Outcomes (KDIGO) guidelines recommend that these patients should perform at least 30 min of moderate physical activity five times per week. Our data show that patients do not comply with these recommendations, regardless of their sex (16.9 min in men and 19.9 min in women). According to Harold Frost’s mechano-stat hypothesis, mechanical usage influences bone mass and geometry [37]. The low level of physical activity among these patients, specifically the low level of moderate/vigorous physical activity, can even induce a decrease in BMD as a consequence of disuse, so the present results of the present study may be influenced by the fact that the women registered longer times of moderate/vigorous activity and lower values of BMD than did men. In fact, renal osteodystrophy is a multifactorial and pervasive disorder of bone and mineral metabolism that is present in individuals with CKD [38]. This disorder leads to a decrease in BMD and increases the risk of bone fracture [39].

The third purpose of the present study was to analyze the relationship between physical activity and biochemical parameters. The results show that light physical activity is negatively related to albumin and total serum protein levels. Considering that increasing the time spent in light physical activity reduces the time available for moderate or vigorous activity and thus makes patients less active, this reduced level of activity therefore induces a reduction in albumin level. Cupisti et al. [27] reported that PD patients with lower levels of physical activity presented lower levels of albumin. It is known that albumin is not simply a marker of malnutrition but also reflects underlying inflammation and comorbidities, particularly cardiac comorbidities, with lower levels of albumin being related to inflammation and mortality [40]. Recent studies showed that survivors had significantly higher plasma albumin values than did none-survivors [6,40]. We can expect that increasing the time in moderate/vigorous physical activity can increase the plasma albumin level. Low total protein levels are related to higher levels of C-reactive protein (CRP), an inflammatory marker and the gold standard for the prediction of morbidity and mortality in CKD patients [41]. The inverse relation between light physical activity and total serum protein level indicates that patients with higher levels of light physical activity are at a higher risk of morbidity and mortality; thus, it is important for these patients to increase the time they spend performing moderate/vigorous activity.

The time spent in moderate/vigorous physical activity was positively related to the weekly Kt/V (urea) and creatinine clearance and negatively related to triglyceride level. According to Wang et al. [40], survivors presented higher weekly Kt/V (urea) values than did none-survivors. Lo et al. [42] reported that lower weekly Kt/V and/or creatinine clearance values are associated with increased mortality. Increasing the time spent in moderate/vigorous activity can eventually lead to beneficial changes in these variables in PD patients.

Higher levels of triglycerides are related to cardiovascular events in the general population [43]. Recently, Wu et al. [43] showed that serum triglyceride level was associated with an increased risk of all-cause and cardiovascular disease-related mortality in PD patients, with a sex difference, since the women demonstrated a significantly higher cardiovascular risk than did men. If the negative relationship between moderate/vigorous physical activity and serum triglycerides is confirmed in future studies, increasing the practice of intense physical activity should clearly be a priority for PD patients.

Greater fat mass was associated with a higher CRP level (*r* = 0.501; *p* = 0.003). This result is consistent with those reported by Majchrzak et al. [24] and Axelsson et al. [44]. Healthy people generally exhibit low levels of CRP. However, it seems that PD patients who exhibit higher levels of adipose tissue are more likely to show increased levels of biomarkers of inflammation, such as CRP.

The present study has several limitations worth considering. Firstly, the sample size was small, and the reason why each patient started PD, the duration of treatment after diagnosis, and the physical activity levels were heterogeneous. However, all applicants were stable and did not have severe comorbidities. Secondly, lifestyle factors, in particular the practice of physical activity/exercise in the months before the study, was not controlled and may possibly have influenced some of the variables. Thirdly, given the cross-sectional nature of this research, we were able to establish associations but not causal relationships. More studies with more homogenous groups are needed to clarify our results, particularly studies on the relationship between physical activity level and BMD.

## 5. Conclusions

The most important finding of the present study is that PD patients who spend more time performing moderate or vigorous physical activity have healthier levels of the different variables studied, except for BMD. More studies are needed to clarify whether BMD actually decreases in response to an increase in the level of physical activity among these patients. If this negative result remains, it will be necessary to find a way to compensate for this unwanted effect through compensatory programs with an osteogenic effect, such as complementary activities with impact.

## Figures and Tables

**Table 1:** Descriptive summary of patients’ demographics, body composition, physical activity, muscle strength and biochemical parameters. Results are expressed as mean ± SD or number of observations (percentage of total).

Data Collected	Results
Demographics (*n* = 34)	
Gender	
Male	21 (62%)
Female	13 (38%)
Age (years)	47.8 ± 13.4
Diabetes Mellitus (%)	25.5
**Body Composition (*n* = 34)**	
Weight (kg)	65.3 ± 11.1
Height (cm)	162.6 ± 10.0
Body Mass Index (kg/m^2^)	24.6 ±3.7
Percentage of Fat Mass (%)	28.5 ± 8.5
Lean Body Mass (kg)	46.1 ± 8.7
Fat Mass (kg)	18.5 ± 7.0
Bone Mineral Content (kg)	1.9 ± 0.4
Bone Mineral Density (g/cm^2^)	1.0 ± 0.1
TBW (kg)	37.1 ± 6.3
%HLBM	81.1 ± 6.4
**Physical Activity (*n* = 31)**	
Sedentary (min)	1161.7 ± 93.5
Light (min)	206.4 ± 91.4
Moderate/Vigorous (min)	16.9 ± 15.2
Steps (n)	4999.2 ± 2477.4
**Muscle Strength (*n* = 32)**	
Knee extensors strength (N·m/kg)	87.8 ± 38.3
Knee flexors strength (N·m/kg)	51.4 ± 21.6
Elbow extensors strength (N·m/kg)	22.0 ± 7.7
Elbow flexors strength (N·m/kg)	37.2 ± 10.3
**Analytical Chemistry (*n* = 34)**	
Serum Albumin (g/dL)	3.9 ± 0.4
Serum total proteins (g/dL)	7.1 ± 0.8
Serum CRP (mg/L)	0.7 ± 0.4
Serum HDL (mg/dL)	46.8 ± 13.9
Serum Triglycerides (mg/dL)	170.7 ± 71.6
Serum Cholesterol (mg/dL)	184.3 ± 31.7
Serum LDL (mg/dL)	104.9 ± 39.7
Serum glucose (mg/dL)	109.4 ± 109.2
Creatinine Clearance (L/1.73 m^2^ weekly)	75.0 ± 35.2
Kt/V urea (weekly)	2.0 ± 0.6
Serum sodium (mmol/L)	137.7 ± 3.8

TBW = total body water; %HLBM = percentage of hydration of lean body mass; CRP = C-reactive protein; HDL = high density lipoprotein; LDL = low density lipoprotein.

**Table 2:** Body composition variables according to gender. Results are expressed as mean ± SD.

	Men (*n* = 21)	Women (*n* = 13)	*p* Value
Weight (kg)	69.1 ± 10.4	59.1 ± 9.6	0.008 ^a^
Height (cm)	168.3 ± 6.9	153.4 ± 6.6	<0.001 ^a^
BMI (kg/m^2^)	24.2 ± 3.6	25.1 ± 3.9	0.486 ^a^
%FM	24.3 ± 6.0	35.5 ± 7.5	<0.001 ^a^
LBM (kg)	51.6 ± 5.6	37.3 ± 4.7	0.001 ^a^
FM (kg)	17.0 ± 6.5	21.2 ± 7.2	0.092 ^a^
BMC (kg)	2.2 ± 0.3	1.5 ± 0.4	0.001 ^a^
BMD (g/cm^2^)	1.1 ± 0.1	0.9 ± 0.1	0.005 ^b^
TBW/Weight (%)	58.8 ± 3.6	54.6 ± 8.3	0.106 ^a^
%HLBM	78.4 ± 4.5	85.4 ± 6.7	0.004 ^a^

^a^—T Student Test *p*-value; ^b^—Mann-Whitney Test *p*-value; BMI = body mass index; %FM = percentage of fat mass; LBM= lean body mass; FM = fat mass; BMC = bone mineral content; BMD = bone mineral density; TBW = total body water; %HLBM = percentage of hydration of lean body mass.

**Table 3:** Differences in Physical Activity and Muscle Strength by gender. Results are expressed as mean ± SD.

	Men (*n* = 21)	Women (*n* = 13)	*p* Value
Physical Activity *			
Sedentary (min/day)	1170.2 ± 107.0	1146.2 ± 64.0	0.107 ^b^
Light (min)	199.0 ± 104.8	220.0 ± 62.5	0.231 ^b^
Moderate/Vigorous (min)	16.9 ± 15.3	19.9 ± 15.1	0.653 ^b^
Steps	4779.8 ± 2711.5	5398.2 ± 2042.4	0.515 ^a^
Muscle Strength **			
Knee extensors strength (N·m/kg)	94.6 ± 42.6	76.8 ± 28.4	0.193 ^a^
Knee flexors strength (N·m/kg)	59.8 ± 21.7	37.8 ± 13.1	0.002 ^a^
Elbow extensors strength (N·m/kg)	26.5 ± 6.0	15.5 ± 4.5	<0.001 ^a^
Elbow flexors strength (N·m/kg)	42.5 ± 9.2	29.3 ± 6.1	<0.001 ^a^

*—Men (*n* = 20); Women (*n* = 11); **—Men (*n* = 19); Women (*n* = 13); ^a^—*p* of the T Student; ^b^—*p* of the Mann-Whitney Test; Data expressed by mean (SD).
